# The versatile application of cervicofacial and cervicothoracic rotation flaps in head and neck surgery

**DOI:** 10.1186/1477-7819-9-135

**Published:** 2011-10-23

**Authors:** Fa-yu Liu, Zhong-fei Xu, Peng Li, Chang-fu Sun, Rui-wu Li, Shu-fen Ge, Jun-lin Li, Shao-hui Huang, Xuexin Tan

**Affiliations:** 1 Department of Oromaxillofacial - Head & Neck Surgery; Department of Oral and Maxillofacial Surgery, School of Stomatology, China Medical University, No. 117, Nanjing Bei Jie, Heping District, Shenyang, Liaoning, 110002, China; 2 Department of Head and Neck, Henan Tumor Hospital, Zhengzhou University, 127 Dongming Road, Zhengzhou, Henan, 450008, China

**Keywords:** cervicofacial flap, cervicothoracic flap, head and neck, reconstruction

## Abstract

**Background:**

The large defects resulting from head and neck tumour surgeries present a reconstructive challenge to surgeons. Although numerous methods can be used, they all have their own limitations. In this paper, we present our experience with cervicofacial and cervicothoracic rotation flaps to help expand the awareness and application of this useful system of flaps.

**Methods:**

Twenty-one consecutive patients who underwent repair of a variety of defects of the head and neck with cervicofacial or cervicothoracic flaps in our hospital from 2006 to 2009 were retrospectively analysed. Statistics pertaining to the patients' clinical factors were gathered.

**Results:**

Cheek neoplasms are the most common indication for cervicofacial and cervicothoracic rotation flaps, followed by parotid tumours. Among the 12 patients with medical comorbidities, the most common was hypertension. Defects ranging from 1.5 cm × 1.5 cm to 7 cm × 6 cm were reconstructed by cervicofacial flap, and defects from 3 cm × 2 cm to 16 cm × 7 cm were reconstructed by cervicothoracic flap. The two flaps also exhibited versatility in these reconstructions. When combined with the pectoralis major myocutaneous flap, the cervicothoracic flap could repair through-and-through cheek defects, and in combination with a temporalis myofacial flap, the cervicofacial flap was able to cover orbital defects. Additionally, 95% patients were satisfied with their resulting contour results.

**Conclusions:**

Cervicofacial and cervicothoracic flaps provide a technically simple, reliable, safe, efficient and cosmetic means to reconstruct defects of the head and neck.

## Background

The variable surgical defects that can result from head and neck operations necessitate a broad range of surgical reconstructions, ranging from primary closures and pedicle flaps to free tissue transfers. According the distribution of blood supply, the pedicle flap can include random flaps and axial flaps. A random blood supply pattern is needed to maintain a wide pedicle[1]. Therefore, many random flaps, such as cheek advancement-rotation flaps and forehead flaps, have poor mobility and are only suitable for reconstructing small defects. Although axial flaps (e.g., trapezius flaps[2] and pectoralis major myocutaneous flaps (PMMF)[3]) can overcome these limitations, they often appear too bulky or large, result in a poor colour match with the recipient site, and sometimes impair the function of donor muscle groups.

In recent years, with the advancement of microsurgical techniques, new flap techniques offer great hope for the future of head and neck reconstruction, including radial forearm flaps, anterolateral thigh free flaps[4], rectus abdominis myocutaneous flaps[5], and latissimus dorsi flaps[6]. However, to optimise the cosmetic and functional outcomes for any given individual surgical wound, the head and neck surgeon must possess a firm grasp of fundamental techniques as well as the ability to use a reconstructive modality that meets the unique demands of each defect, as ascertained through a thorough defect analysis[7]. Furthermore, not all patients are suitable candidates for free flaps. The surgeon should select the proper reconstruction methods according to the patients' general body states, the match of the texture and colour of the flap with the recipient region, the patient's body position, medical complications, whether two operation sites are needed and the surgeon's clinical experience.

Cervicofacial and cervicothoracic rotation flaps are two variants of the random flap, both with wide pedicles. They are time-honoured methods in head and neck reconstruction that can provide excellent skin colour, thickness and texture match, with cosmetically acceptable scars and minimal morbidity. Therefore, they are adapted to fit many defects of the face, cheek, parotid region, periorbital region, auricle and neck. Particularly in certain high-risk patients, such as the very old, those with many systemic diseases, or who for any reason cannot tolerate a long operative time, they seem to be the preferred option. Despite these advantages, cervicofacial and cervicothoracic rotation flaps have received scarce attention in the literature. In this article, we present our experience in using cervicofacial and cervicothoracic rotation flaps to expand the awareness and promote the application of this useful system of flaps.

## Methods

### Patients

Twenty-one consecutive patients who underwent repairs of a variety of defects of the head and neck with cervicofacial or cervicothoracic flaps in our hospital from 2006 to 2009 were included in this study. Patients' charts were reviewed for patient demographic information; defect location; pathologic diagnosis; comorbid disease, including hypertension, cardiac disease and diabetes mellitus; coincident procedures, such as neck dissection; the type of flap used and the associated flap; the length of hospitalisation; complications; and aesthetic outcome. Local Ethical Committee approval was granted for the use of surgical trimming, and informed consent was also obtained from the patients before surgery.

### Surgical Technology

**Figure 1:**
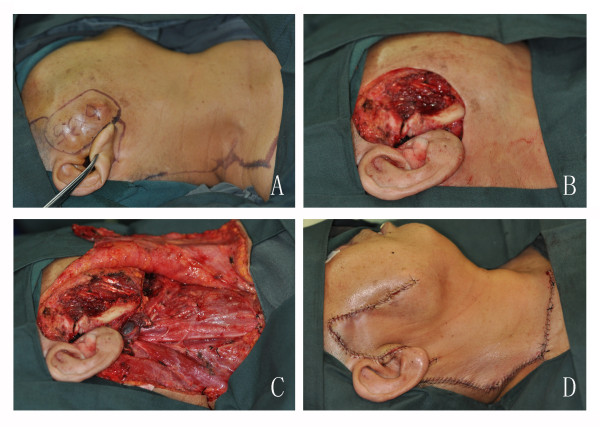
**The surgical technique of the cervicofacial flap**. A. The outline for the resection of the tumour and cervicofacial flap design. B. The defect following resection of the tumour. C. The flap was raised, and the neck dissection was completed. D. The donor site was closed primarily.

The incision outline for the resection of tumours and cervicofacial flap design are shown in Figure 1A. The incision begins from the posterolateral aspect of the resection margins (Figure 1B). By carrying the incision below the lobule and then up to the mastoid tip, it can then be dropped into the neck along the anterior edge of the trapezius muscle, the lateral third of the clavicle, and extending into the pectoral region. The incision is performed in a staged fashion, with continual reassessment of the arc of rotation and the ability of the flap to fill the defect until a tension-free closure can be accomplished. The incisions include potential avenues for a back cut along natural skin creases. If the base of the inferior limit of the incision is up to the clavicle, the flap is named a cervicofacial flap (Figure 1C). If the base of the inferior limit is down to the clavicle, then the flap is named a cervicothoracic flap. The flaps were raised superficial to the superficial musculoaponeurotic system (SMAS) and the parotidomasseteric fascia, deep to the platysma, and then transferred into the outer skin defect and sutured (Figure 1D).

### Statistical analysis

The association of cervicofacial or cervicothoracic flap complications with comorbidities was analysed using a χ2 test with the software SPSS 11.0. The evaluation of postoperative appearance was performed according to the method described by Peng et al.[8]. A score of 7 points was rated as excellent, 6 and 5 points good, and fewer than 4 points poor. A patient with a score of no less than 5 points was considered to have an acceptable appearance.

## Results

**Figure 2:**
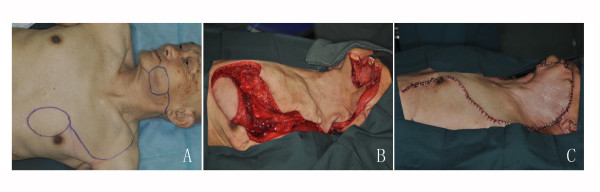
**The combination of cervicothoracic rotation flaps and PMMF**. A. The outline of the resection of the tumour and the design of the combination of cervicothoracic flaps and PMMF. B. The cervicothoracic flap and PMMF were raised after tumour resection. C. PMMF was used to reconstruct the mucosa, and the cervicothoracic flap was used to cover the defect. The donor site was closed primarily.

**Figure 3:**
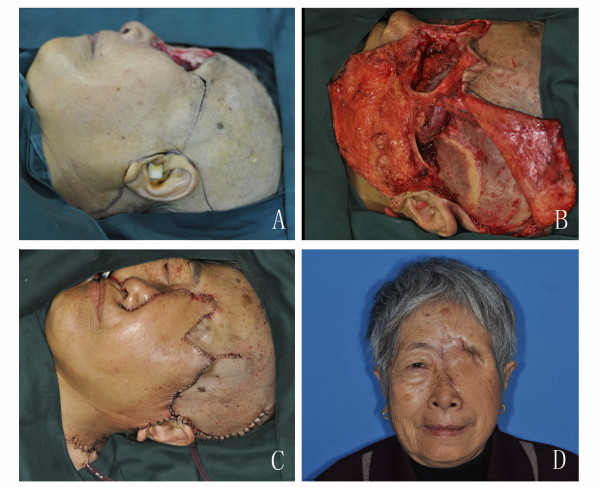
**The combination of cervicofacial rotation flap and TMF**. A. The design of the TMF and cervicofacial flap after the tumour was resected. B. The TMF was rotated and advanced to replenish the orbital defect, and the cervicofacial flap was raised. C. The orbital defect was closed with no significant contour deformity. D. Result 2 months postoperatively.

From a review of medical records, 21 patients were identified whose surgical defects were reconstructed with cervicofacial or cervicothoracic rotation flaps (Table 1). The patients ranged in age from 46 to 87 years, with a mean age of 64.5 years. These patients included 12 men and 9 women. The patients exhibited a myriad of comorbidities, including 9 patients with hypertension, 5 patients with diabetes mellitus, 1 patient with cardiac disease, and 3 patients with two conditions. The most frequently encountered surgical defect site was the cheek (n = 7), and the most common histologic diagnosis was squamous cell cancer (n = 10). The size of the defects ranged from 1.5 cm × 1.5 cm to 16 cm × 7 cm. Ten patients were reconstructed with cervicofacial rotation flaps, and 11 patients were reconstructed with cervicothoracic flaps. Neck dissections were performed in 12 cases (57%), ranging from selective nodal dissections to radical neck dissections. There were 4 patients who suffered through-and-through cheek defects, with skin defects ranging from 4 cm × 4 cm to 6 cm × 4 cm and cheek mucosal defects ranging from 2 cm × 1.5 cm to 4 cm × 3 cm. They were all reconstructed with combined cervicothoracic rotation flaps and PMMF (Figure 2). The PMMF were used to repair the cheek mucosa, and the cervicothoracic flaps were used to repair the cheek skin. All of the chest donation sites were able to be closed primarily. In 3 patients with deep defects, pedicled pectoralis myofascial flaps (PMF) or temporalis myofacial flaps (TMF) were used for tissue bulk (Figure 3).

**Table 1:** Twenty-one consecutive patients undergoing resection and reconstruction with cervicofacial or cervicothoracic flap.

patient	sex	Age(y)	Defect Location	Pathologic diagnosis	Comorbid disease	Size (cm)		Neck dissection	Flap used	Associated flap	length of postoperative hospitalization(d)	complication	Acceptable appearance
												
						Skin	Mucosa						
1	F	59	face	BCC		3 × 3		-	Cervicofacial	-	11	-	Yes
2	M	74	parotid	AC	Hy	3 × 2		SND	Cervicothoracic	-	10	-	Yes
3	M	78	cheek	SCC		6 × 4	4 × 3	MRND	Cervicothoracic	PMMF	12	-	Yes
4	F	87	parotid	MC	Hy + Dm	5 × 4		-	Cervicofacial	-	10	-	Yes
5	M	46	face	FSa		1.5 × 1.5		-	Cervicofacial	-	7	-	Yes
6	M	56	cheek	SCC		5 × 5	3.5 × 3	RND	Cervicothoracic	PMMF	10	-	Yes
7	F	56	parotid	SCC	Dm	5 × 3		MRND	Cervicothoracic	-	9	-	Yes
8	M	74	parotid	AC	Cd	6 × 5		-	Cervicothoracic	-	8	-	Yes
9	M	50	face	FSa		3 × 3		-	Cervicofacial	-	9	-	Yes
10	F	65	fossa orbitalis	SCC	Hy	6 × 4		-	Cervicofacial	TMF	7	-	Yes
11	F	68	cheek	SCC	Hy + Dm	4 × 4		SND	Cervicofacial	-	8	-	Yes
12	M	53	submaxillary region	ACC		2 × 2		SND	Cervicofacial	-	12	-	Yes
13	F	68	cheek	SCC	Hy	6 × 5	2 × 1.5	RND	Cervicothoracic	PMMF	20	epidermolysis	No
14	M	48	face	SCC	Dm	6 × 6		-	Cervicothoracic	-	23	necrosis of the distal tip	Yes
15	F	51	Cheek	Am		16 × 7		-	Cervicothoracic	PMF	8	-	Yes
16	F	64	cheek	SCC	Dm + Hy	4 × 4	2 × 2	SND	Cervicothoracic	PMMF	15	epidermolysis	Yes
17	M	78	neck	SCC	Hy	7 × 5		RND	Cervicofacial	-	7	epidermolysis	Yes
18	F	77	cheek	SCC	Hy	3 × 3		SND	Cervicothoracic	-	10	-	Yes
19	M	78	Submental region	BCC	Hy	5 × 3		SND	Cervicothoracic	-	8	necrosis of the distal tip	Yes
20	M	53	parotid	MC		7 × 6		RND	Cervicofacial	-	8	-	Yes
21	M	72	fossa orbitalis	BCC		5 × 4		-	Cervicofacial	TMF	7	-	Yes

ACC: adenoid cystic carcinoma. SCC: squamous cell carcinoma. BCC: basal cell carcinoma. MC: myoepithelial carcinoma. FSa: fibrosarcoma. AC: adenocarcinoma Mc: mucoepidermoid carcinoma. Am: ameloblastoma.

Hy: Hypertension. Dm: diabetes mellitus. Cd: cardiac disease.

TMF: temporalis myofacial flap. PMF: pectoralis myofascial flap. PMMF: pectoralis major myocutaneous flap

Patients were hospitalised for 7 to 23 days postoperatively, with a mean hospital stay of 10.4 days. The most common surgical complication was epidermolysis of the distal skin flap (3 cases, 14.3%), and although two patients (9.5%) developed full-thickness necrosis of the distal flap tip, after 10-20 days, both patients recovered gradually. No patients experienced a total flap loss. The vast majority (95%) of patients were satisfied with the contour results of their reconstructions.

## Discussion

Large defects of the head and neck present a significant reconstructive challenge to surgeons, especially when simultaneous parotidectomy and neck dissection is required. Although numerous options can be used, including primary closure, skin grafts, healing by secondary intention, pedicled flaps, and free flaps, all of these techniques have their limitations. The rotation of locoregional tissue remains a mainstay of reconstruction in these anatomic regions. Because it can provide an excellent skin colour and texture match, requires no donor region, reduces the surgical risk to high-risk patients, such as those with complications of hypertension, diabetes, or old age, the random flap is widely used to repair small defects of the head and neck. However, for larger defects, the random flap is of very limited utility. Because of their technical similarities to the random flap, the cervicofacial and cervicothoracic flaps share in its advantages; however, they can also be applied in the reconstruction of large defects.

In 1960, Conley used laterally-based cervical and thoracic rotation flaps to reconstruct wounds of the lateral neck[9]. The closure of skin defects of the face with cervicopectoral rotation flaps was originally reported by Garrett et al. in 1966[10]. The name "cervicofacial flap" was first used by Kaplan in 1978 in a report of the versatility of this flap for the coverage of defects following the removal of cancers of the head and neck. It requires no delay procedures and yields excellent results in terms of both function and appearance. The name "cervicothoracic flap" was coined in the same report. The author used this flap to treat developed chondronecrosis of the thyroid cartilage[11].

In more recent years, the two flaps have been employed and refined by many surgeons and have yielded excellent results. Cook et al. delineated the goals of mid-facial and cheek reconstructions. In addition to providing an excellent skin colour, texture, and thickness match, the tissue should be flexible, minimise distortion of the eye and upper lip, preserve facial movement, and prevent ectropion[12]. The cervicofacial and cervicothoracic flaps meet all of these criteria. In a sense, they are the simplest method of providing soft tissue coverage for the protection of deeper vital structures, such as the facial nerve, mandible, and carotid artery.

In our series, defects ranging from 1.5 cm × 1.5 cm to 7 cm × 6 cm were successfully reconstructed with cervicofacial flaps, and defects ranging from 3 cm × 2 cm to 16 cm × 7 cm were successfully reconstructed with cervicothoracic flaps. The upper boundary of reconstruction with this method can reach to the supraorbital margin, the medial boundary can reach to the median line, and the outer boundary can reach to the pre-auricular or post-auricular region. Without the use of microsurgical techniques, the operations were simpler, and the operative time was shortened significantly.

Moore et al. reported that in their cervicofacial flap and cervicothoracic flap series, 11 patients (31%) experienced some form of wound complication, most often manifested as epidermolysis of the distal skin flap (8/35, 23%), and 3 patients (9%) developed full-thickness necrosis of the distal flap tip[7]. Our data included 3 cases of epidermolysis (3/21, 14.3%) and two cases of distal tip necrosis (2/21, 9.5%). We believe that our lower complication rate may be the results of some methods that we practice in harvesting the flaps. First, we are careful to protect the superficial cervical fascia surrounding the platysma and thus preserve the capillary network in the fascia. Second, during the flap harvest, we often elevated the superficial veins with the flap, although they were ligated on both sides. We believe that the residual vein can be helpful in re-establishing blood circulation. Finally, when resecting the tumours, we take care not to produce excessive thinning of the skin surrounding the defect. Thus, the distal aspect of the flap can be provided with more subcutaneous tissue, which could supply a rich network of subdermal anastomoses.

There was no significant association between the overall complication rate and hypertension (χ2 = 3.697, p = 0.055) or diabetes mellitus (χ2 = 0.948, p = 0.330). This is consistent with the conclusion of Moore et al.[7]. However, when epidermolysis and distal necrosis were analysed independently, we found that epidermolysis was correlated with hypertension (χ2 = 4.667, p = 0.031) and that diabetes mellitus also exhibited a non-significant trend toward a greater complication rate. Among 13 hypertensive patients with free flap reconstructions in our hospital, two (15.4%) patients experienced the complication of total flap loss. Although the overall complication rate of our cervicofacial and cervicothoracic flaps in hypertensive patients (4/9, 44.4%) was higher than with free flaps, the impact of these complications on aesthetic outcomes was often slight, with the epidermolysis and distal necrosis recovering gradually. Furthermore, we suggest that the low complication rate of the free flaps may be partially due to strict indications. Hakim et al. reported 6 patients with dorsally pedicled platysma cervicofacial rotation flap-reconstructed orbital and cheek defects with no significant complications[13]. They believed that the dorsal pedicle and platysma would enhance the blood supply to the flaps. In our series, cervicofacial flaps were all medially pedicled. Although 1 patient in our series experienced epidermolysis of the distal tip of the flap (1/10, 10%), the scar was inconspicuous, with an incision following the pre-auricular, anterior edge of the trapezius muscle and a natural neck skin crease. The use of deep planes for cervicofacial flaps has been advocated recently, mainly because of greater reliability with excellent vascularity[1]. Tan, in the largest study to date of deep-plane cervicofacial flaps, reports a 6% tip necrosis rate (1/18)[1], consistent with our finding of 10% in traditional subcutaneous cervicofacial flaps (1/10). These results are too similar to advocate for one approach over the other, however, we have avoided the deep-plane methods because they are more time consuming, pose a greater risk of facial nerve damage and require very experienced surgeons for success[14].

Cervicofacial and cervicothoracic flaps are not suitable for the repair of all possible defects of the head and neck. They are primarily useful in relatively superficial defects because they are too thin to provide the necessary bulk of tissue for deeper repairs. However, they have also exhibited their versatility in this respect. In this study, 4 patients with through-and-through cheek defects required the reconstruction of mucosa and cheek skin simultaneously. The single forearm flap is too thin for this application, and the anterolateral thigh free flap or PMMF would seem to be too thick after being folded. Therefore, we used the combination of cervicothoracic flaps and PMMF with good results (Figure 2). Moore et al. reported that they performed a PMF combined with cervicothoracic flaps to supplement additional soft tissue in the case of an extended radical neck dissection and carotid artery replacement[7]. In contrast with the PMF, the PMMF requires the harvest of some skin, which might make it more difficult to close the donor site on the chest; however, in our series, the defects from 7 cm × 6 cm to 10 cm × 7 cm on the chest were all able to be closed primarily. There were also 2 patients with orbital defects for whom we used temporalis myofacial flaps to replenish the dead space and cervicofacial flaps to cover the surface, thus not only shortening the operation times but also reducing the postoperative complications (Figure 3).

## Conclusions

Because cervicofacial and cervicothoracic flap techniques are anatomically sound, technically simple, reliable and safe, we believe that they are useful methods for the reconstruction of surgical defects of the head and neck and will more frequently attract the attention of surgeons in the future.

## Competing interests

The authors declare that they have no competing interests.

## Authors' contributions

LF and CS formulated the manuscript. ZX and PL carried out the statistical analysis of studies. CS, RL, JL, SG, SH and XT participated the design and the performance of operation. All authors read and approved the final manuscript.
